# Identifying conserved protein complexes between species by constructing interolog networks

**DOI:** 10.1186/1471-2105-14-S16-S8

**Published:** 2013-10-22

**Authors:** Phi-Vu Nguyen, Sriganesh Srihari, Hon Wai Leong

**Affiliations:** 1 Department of Computer Science, National University of Singapore, Singapore 117590; 2 Institute for Molecular Bioscience, The University of Queensland, St. Lucia, QLD 4072, Australia

## Abstract

**Background:**

Protein complexes *conserved *across species indicate processes that are *core *to cellular machinery (*e.g*. cell-cycle or DNA damage-repair complexes conserved across human and yeast). While numerous computational methods have been devised to identify complexes from the protein interaction (PPI) networks of individual species, these are severely limited by noise and errors (false positives) in currently available datasets. Our analysis using human and yeast PPI networks revealed that these methods missed several important complexes including those conserved between the two species (*e.g*. the MLH1-MSH2-PMS2-PCNA mismatch-repair complex). Here, we note that much of the functionalities of yeast complexes have been conserved in human complexes not only through sequence conservation of proteins but also of critical *functional domains*. Therefore, integrating information of domain conservation might throw further light on conservation patterns between yeast and human complexes.

**Results:**

We identify conserved complexes by constructing an *interolog network *(IN) leveraging on the *functional conservation *of proteins between species through *domain conservation *(from Ensembl) in addition to sequence similarity. We employ 'state-of-the-art' methods to cluster the interolog network, and map these clusters back to the original PPI networks to identify complexes conserved between the species. Evaluation of our IN-based approach (called COCIN) on human and yeast interaction data identifies several additional complexes (76% recall) compared to direct complex detection from the original PINs (54% recall). Our analysis revealed that the IN-construction removes several non-conserved interactions many of which are false positives, thereby improving complex prediction. In fact removing non-conserved interactions from the original PINs also resulted in higher number of conserved complexes, thereby validating our IN-based approach. These complexes included the mismatch repair complex, MLH1-MSH2-PMS2-PCNA, and other important ones namely, RNA polymerase-II, EIF3 and MCM complexes, all of which constitute core cellular processes known to be conserved across the two species.

**Conclusions:**

Our method based on integrating domain conservation and sequence similarity to construct interolog networks helps to identify considerably more conserved complexes between the PPI networks from two species compared to direct complex prediction from the PPI networks. We observe from our experiments that protein complexes are not conserved from yeast to human in a straightforward way, that is, it is not the case that a yeast complex is a (proper) sub-set of a human complex with a few additional proteins present in the human complex. Instead complexes have evolved multifold with considerable re-organization of proteins and re-distribution of their functions across complexes. This finding can have significant implications on attempts to extrapolate other kinds of relationships such as synthetic lethality from yeast to human, for example in the identification of novel cancer targets. *Availability*: http://www.comp.nus.edu.sg/~leonghw/COCIN/.

## Background

Complexes of physically interacting proteins form fundamental units responsible for driving key biological processes within cells. Even in the simple model organism *Saccharomyces cerevisae *(budding yeast), these complexes are composed to several protein subunits that work in a coherent fashion to carry out cellular functions. Therefore a faithful reconstruction of the entire set of complexes (the 'complexosome') from the set of physical interactions (the 'interactome') is essential to understand their organisation and functions as well as their roles in diseases [1-4].

In spite of the significant progress in computational identification of protein complexes from protein interaction (PPI) networks over the last few years (see the surveys [1,2]), computational methods are severely limited by noise (false positives) and lack of sufficient interactions (*e.g*. membrane-protein interactions) in currently available PPI datasets, particularly from human, to be able to completely reconstruct the complexosome [1,2]. For example, several complexes involved in core cellular processes such as cell cycle and DNA damage response (DDR) are not present in a recent (2012) compendium of human protein complexes (http://human.med.utoronto.ca/) assembled solely by computational identification of complexes from high-throughput PPIs[5]; a web-search (as of Feb 2013) in this compendium for BRCA1 does not yield any complexes even though BRCA1 is known to participate in three fundamental complexes in DDR *viz*. BRCA1-A, BRCA1-B and BRCA1-C complexes [6-8]. A possible reason for missing these complexes is the lack of sufficient PPI data required for identifying them even using the best available algorithms. But, the authors of this compendium note that many human complexes appear to be ancient and slowly evolving - roughly a quarter of the predicted complexes overlapped with complexes from yeast and fly, with half of their subunits having clear orthologs [5]. Therefore, it is useful to devise effective computational methods that look for evidence from evolutionary conservation to complement PPI data to reconstruct the full set of complexes.

In the attempt to integrate evolutionary information with PPI networks, Kelley *et al. *[9] and Sharan *et al. *[10] devised methods to construct an *orthology graph *of conserved interactions from two species, which in their experiments were yeast (*S. cerevisae*) and bacteria (*H. pylori*), using a sequence homology-based (using BLAST E-score similarity) mapping of proteins between the species. Dense sub-graphs induced in this orthology graph represented putative complexes conserved between the two species. The complexes so-identified were involved in core cellular processes conserved between the two species - e.g. those in protein translation, DDR and nuclear transport. Van Dam and Snel (2008) [11] studied rewiring of protein complexes between yeast and human using high-throughput PPI datasets mapped onto known yeast and human complexes. From their experiments, they concluded that a majority of co-complexed protein pairs retained their interactions from yeast to human indicating that the evolutionary dynamics of complexes was not due to extensive PPI network rewiring within complexes but instead due to gain or loss of protein subunits from yeast to human. Hirsh and Sharan [12] developed a protein evolution-based model and employed it to identify conserved protein complexes between yeast and fly, while Zhenping *et al. *[13] used integer quadratic programming to align and identify conserved regions in molecular networks. Marsh *et al. *[14] integrated data on PPI and structure to understand mechanisms of protein conservation; they found that during evolution gene fusion events tend to optimize complex assembly by simplifying complex topologies, indicating genome-wide pathways of complex assembly.

### Integrating domain conservation

**Figure 1:**
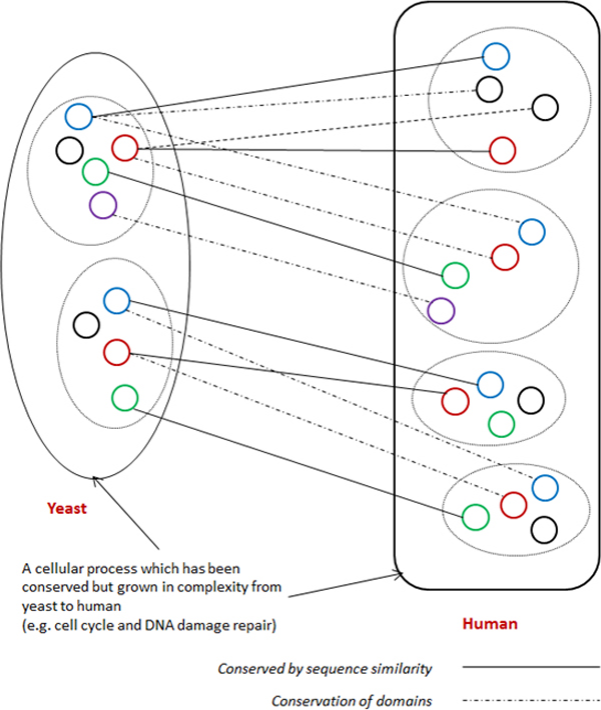
**Conservation of complexes between yeast and human**. Many proteins in yeast have either 'split' into multiple proteins or fused into common proteins in human during evolution. This mechanism is a result of selecting optimal protein assemblies [14] thereby resulting in multi-fold expansion of complexity in human. In order to capture these conservation mechanisms it is necessary to integrate domain along with PPI information.

Inspired from these works, here we devise a novel computational method to identify *conserved complexes *and apply it to yeast and human datasets. A crucial point we note on the conservation from yeast to human is that many cellular mechanisms, though conserved, have in fact evolved many-fold in complexity - for example, cell cycle and DDR. Consequently, while several proteins in these mechanisms are conserved by sequence similarity (e.g. RAD9 and hRAD9), there are others that are unique (non-conserved) to human (e.g. BRCA1); see Figure 1. These non-conserved proteins perform similar functions (e.g. cell cycle and DDR) as their conserved counterparts, but do not show high sequence similarity to any of the yeast proteins. A deeper examination reveals that these proteins in fact contain *conserved functional domains *- for example, the BRCT domain which is present in yeast RAD9 and human hRAD9 is also present in the non-conserved human BRCA1 and 53BP1; all of these play crucial roles in DDR [15]. Similar structure can be seen in the case of RecQ helicases - several helicase domains are conserved from the yeast SGS1 to human BLM and WRN, but there are three helicases RECQ1,4,5 which are unique to human that also contain these helicase domains [16]. Therefore, integrating information on *functional conservation*, mainly through *domain conservation*, can help to identify considerably more (functionally) conserved complexes than mere sequence similarity, thereby throwing further light on the conservation patterns of complexes in particular and cellular processes in general.

In order to achieve this, simple BLAST-based scores as used in earlier works [9-13] to measure homology between yeast and human proteins do not suffice. Here, we integrate multiple databases including Ensembl [17] and OrthoMCL [18] to build homology relationships among proteins; these databases use a variety of information to construct *orthologous groups *among proteins including checking for *conserved domains*. The integration of these databases generates *many-to-many *correspondence between yeast and human proteins instead of the predominantly one-to-one correspondence obtained by from BLAST-based similarity.

We devise a novel computational method to construct an *interolog network *using domain information along with PPI conservation between human and yeast. Next, we identify dense clusters within the interolog network using current 'state-of-the-art' PPI-clustering methods (as against traditional clustering methods used in [9,10]). These clusters when mapped back to the PPI networks reveal conserved dense regions, many of which correspond to conserved complexes.

Our experiments here reveal that,

(i) integrating domain information generates many valuable interactions from the many-to-many ortholog relationships in the interolog network, thereby enhancing its quality;

(ii) interolog network also reduces false-positive interactions by accounting for conserved PPIs;

(iii) our interolog network construction aids clustering algorithms to identify far more conserved complexes than direct clustering of the individual PPI networks; and

(iv) many of these conserved complexes are involved in core cellular processes such as cell cycle and DDR throwing further light to the conservation of these cellular processes.

We call our method **COCIN **(COnserved Complexes from Interolog Networks).

## Methods

### Constructing the interolog network

Given two PPI networks from two species S_1 _and S_2_, and the homology information between proteins of the two networks, we construct an *interolog network *G_I _as follows. The two PPI networks are represented as G_1_(V_1_, E_1_) and G_2_(V_2_, E_2_), and the homology relationship between the proteins is governed by a *many-to-many correspondence *θ: V_1 _→ V_2_. The interolog network is defined as G_I_(V_I_, E_I_), where V_I _= {v_I_= {p, q} | p∈V_1_, q∈V_2_, and (p, q)∈ θ}, and E_I_= {(v_I_, v'_I_) | v_I =_{p,q}; v'_I=_{r,s}; (p, r)∈E_1 _and (q,s)∈E_2_}.

**Figure 2:**
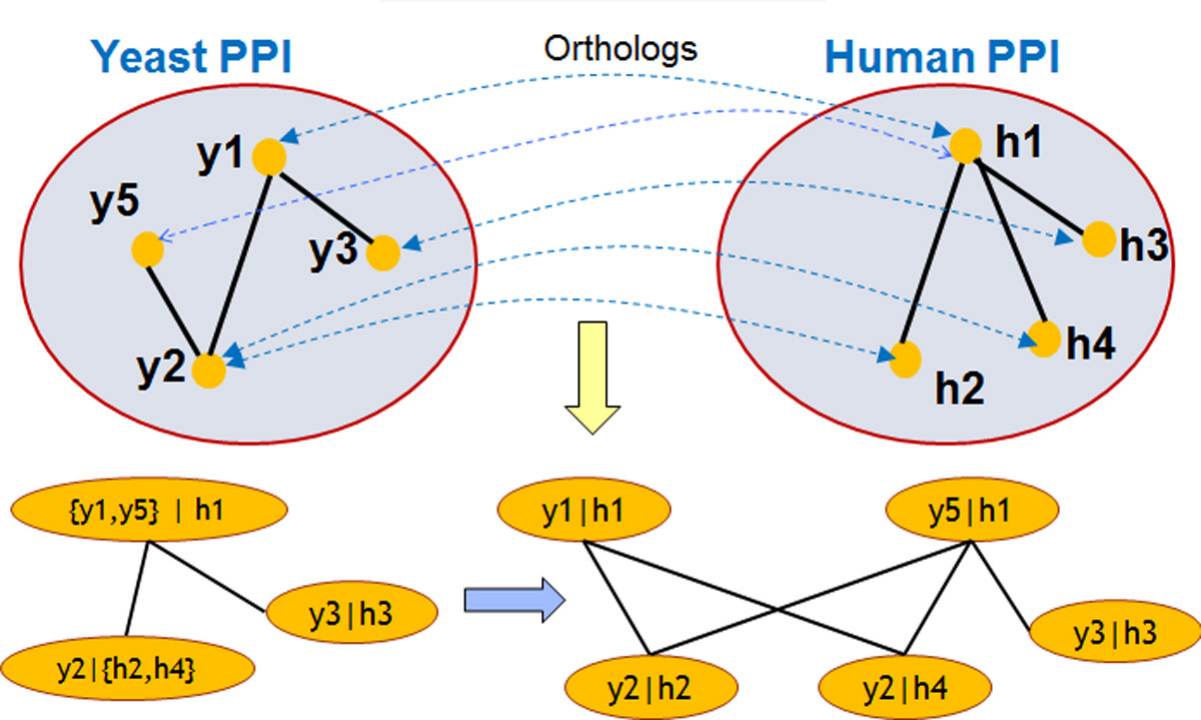
**Construction of the interolog network - a simplified example**. Our interolog network constructing integrates PPI and domain conservation information to generate a network that is conducive for clustering algorithms to identify considerably more conserved complexes compared to direct clustering of the original PPI networks from species.

Each node in the interolog network represents a *pair of homologous proteins*, one from each species. Each edge in the interolog network represents an interaction that is *conserved *in both species (interolog). However, if a protein p ∈ V_1 _can be orthologous to multiple proteins x ∈ V_2 _and x ∈ V_2, _then we add two vertices to G_I _namely {p, x} and {p, y}, and add an edge between two vertices. Doing so integrates the many-to-many relationships obtained due to domain conservation into the interolog network. Figure 2 below gives a simple example of this network-construction.

Any connected sub-network in this interolog network can be mapped back to conserved sub-networks in the two PPI networks, and this is similar to the orthology graph method introduced by Kelley *et al. *[9] and Sharan *et al. *[10]. However, one unique advantage of our interolog network offers is that we can infer a *collection *of homologous complexes between the species. This property is highly relevant for identifying conserved complexes between yeast and human (revisit Figure 1).

In order to achieve this, we integrate multiple databases including Ensembl [17] and OrthoMCL [18] to build our homology relationships among proteins; these databases use a variety of information to construct orthologous groups among proteins including checking for conserved domains.

### Clustering the interolog network and detection of conserved complexes

We identify dense clusters in the interolog network to detect conserved complexes between the two species. To do this, we tested a variety 'state-of-the-art' PPI network-clustering methods, and found the following three to perform the best - CMC (Clustering by merging Maximal Cliques) by Liu *et al. *[19], MCL (Markov Clustering) by van Dongen [20] and HACO (Hierarchical Clustering with Overlaps) by Wang *et al. *[21]. The comparative assessment of these methods has been confirmed with earlier works [1,2,22-24].

CMC operates by first enumerating all maximal cliques in network, and ranks them in descending order of the weighted interaction density. It then iteratively merges highly overlapping cliques to identify dense clusters in the network. MCL simulates a series of random paths (called a flow) and iteratively decomposes the network into a number of dense clusters. HACO performs hierarchical clustering by repeatedly identifying smaller dense clusters and merging these into larger clusters. HACO has an advantage over the traditional hierarchical clustering because it allows for overlaps (protein-sharing) among the clusters.

Upon finding dense clusters in the interolog network, we map back these clusters to sub-networks within the two PPI networks to identify conserved complexes.

### Building a benchmark dataset for conserved protein complexes

Due to lack of benchmark datasets of conserved protein complexes between human and yeast in the literature, we built our own "gold standard" conserved dataset as follows. Using currently available datasets of manually curated protein complexes of human and yeast, we selected pairs of complexes that shared significant fraction of (homologous) proteins.

For measuring the conservation level of a given complex pair {C_1_, C_2_}, where C_1 _belongs to species S_1 _and C_2 _belongs to species S_2_, we adopted the following *Multi-set Jaccard score*:

*Multi-set Jaccard score*: Let G_C1 _and G_C2 _be the collections of ortholog groups in complexes C_1 _and C_2_, respectively. For any group *g_i_*∈*Gc_i_*(*i *= 1, 2), let *I_Ci _*represent the multiplicity of the group *g_i _*in complex C_i_, which essentially is the number of paralogs within the group. Multi-set Jaccard score is then given by:

$$MSJ\left(C_{1},C_{2}\right)=\frac{\underset{g_{i}\in \left(G_{C1}\cup G_{C2}\right)}{\sum }\text{min}\left({\text{I}}_{C_{1}}\left(g_{i}\right),{\text{I}}_{C_{2}}\left(g_{i}\right)\right)}{\underset{g_{i}\in \left(G_{C1}\cup G_{C2}\right)}{\sum }\text{max}({\text{I}}_{C_{1}}\left(g_{i}\right),{\text{I}}_{C_{2}}\left(g_{i}\right))},$$

**Figure 3:**
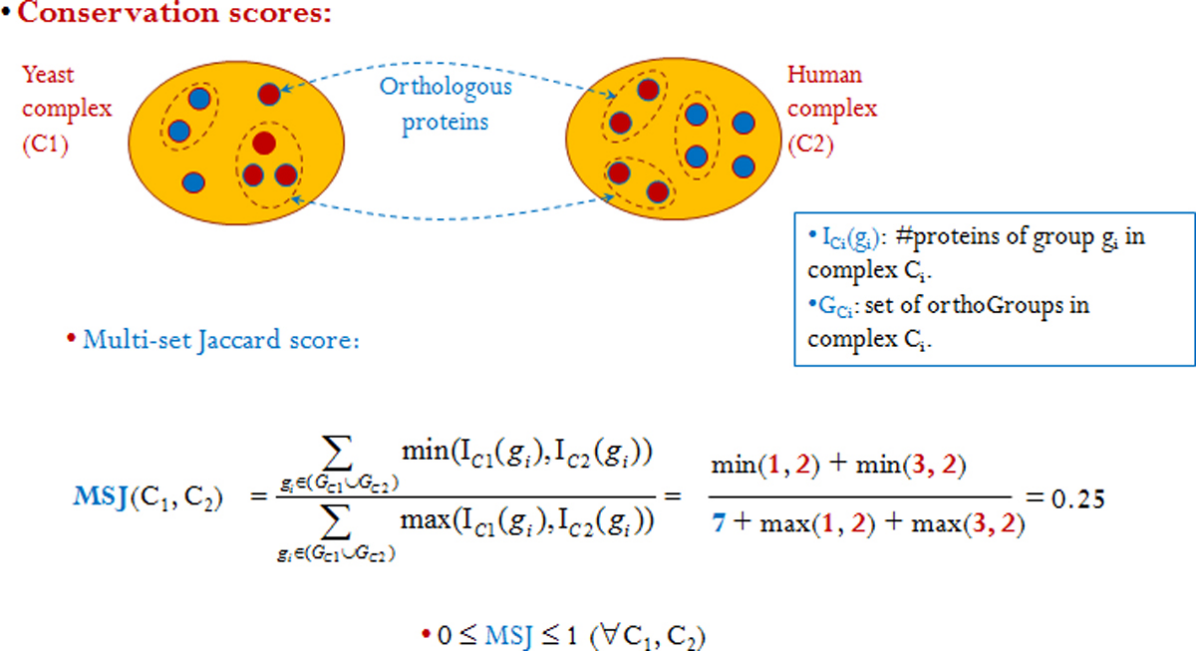
**Conservation scores for building benchmark complex datasets**. We generate a "gold standard" conserved complexes dataset to test our method. We use two scores here - the Jaccard score for orthologous groups and multi-set Jaccard score.

There are often duplication of genes (paralogs) within complexes and clusters. Therefore, MSJ takes into account the multiplicity of the groups and does a more conservative and accurate estimation of the conservation between C_1 _and C_2_. See Figure 3 for an illustration.

We selected pairs of complexes that show MSJ ≥ 50% (see result section for details).

## Results

### Preparation of experimental data

We combined multiple PPI datasets to enhance the coverage of our interactome. We collected PPIs from IntAct [25] (version November 13, 2012) and Biogrid [26] (versions 3.2.95 and 3.2.89) databases for yeast; and from Biogrid [26] and HPRD [27] (Release 9, 2010) for human. Table 1 and 2 summarise these datasets.

**Table 1:** Properties of yeast physical PPI datasets

Database	# proteins	# (non self and duplicated) interactions
IntAct (version Nov 13, 2012)	5276	18834

Biogrid (version 3.2.95, Nov 30, 2012)	5886	73923

IntAct ∪Biogrid	6332	83777

IntAct∩Biogrid	4620	8930

ICDScore(IntAct ∪ Biogrid)	5239	71636

**Table 2:** Properties of human physical PPI datasets

Database	# proteins	#interactions
HPRD (Release 9, 2010)	9617	39184

Biogrid (April 25, 2012)	12515	59027

HPRD ∪ Biogrid	13624	76719

HPRD ∩ Biogrid	8615	21491

ICDScore(HPRD ∪ Biogrid)	8521(EntrezID)	61868

ICDEnrich(HPRD ∪ Biogrid)	9764 (EntrezID)	192053 (EntrezID)

Yeast curated complexes were gathered from Wodak database (CYC2008) [28] and human curated complexes from CORUM (version 09/2009) [29]; these form our *benchmark *complex datasets (details in Table 3). We used Ensembl [17] and OrthoMCL [18] for the homology mapping between human and yeast proteins.

**Table 3:** Properties of manually curated protein complex datasets

Databases	# complexes
**Wodak**[28] yeast complexes (CYC 2008)	**149 **with size>3 (36.5%)
	
	Total: 408

**CORUM **[29] human complexes (September 2009)	**722 **with size>3 (39.1%)
	
	Total: 1843

### Criteria for evaluating predicted complexes

For a predicted complex *C_i _*of one species and a manually curated (benchmark) complex *B_j_*, we used Jaccard score based on collections of complex proteins: $J\left(C_{i},B_{j}\right)=\frac{\left|C_{i}\cap B_{j}\right|}{\left|C_{i}\cup B_{j}\right|}$, which considers *C_i _*a correct prediction for *B_j _*if *J*(*C_i_, B_j_*)≥*t*, a *match threshold*. We chose *t *= 0.50 in our experiments as suggested by earlier works [19,22]. *C_i _*is then referred to as a *matched prediction *or *matched predicted complex*, and *B_j _*is referred to as a *derived benchmark complex*.

Based on this, *precision *is computed as the fraction of predicted complexes matching benchmark complexes, and the *recall *is computed as the fraction of benchmark protein complexes covered by our predicted complexes. A correctly predicted complex is also checked against our "gold standard" testing dataset to see if it is a conserved complex, in which case the derived complex is a *derived conserved complex*.

### Results of complex detection using interolog network (IN)

Table 4 summarizes the interolog network constructed from yeast and human PPIs. We map back each predicted cluster from the IN to the original PPI networks to predict conserved complexes between the two species.

**Table 4:** Properties of the interolog network constructed from yeast and human PPIs

# Mapped nodes using orthology	2470
# Interologs	6133

Size of biggest connected component	2434 nodes, 6112 edges

#Other connected components	16 (size from 2-3)

Firstly, we compared the results of complex detection from COCIN with direct clustering of the original PPI networks using CMC, HACO and MCL as shown in Tables 5 and 6. Interestingly, we observed that COCIN, which employs CMC, HACO and MCL for clustering the interolog network, yielded a better recall than these methods on the original PPI networks. Further, because IN capitalises on the existence of interactions in both PPI networks (that is, conservation of interactions), the number of noisy dense clusters in COCIN is considerably reduced thereby enhancing its precision.

**Table 5:** Comparisons of different methods on yeast data.

Method	# Predicted complexes	# Matched predictions	Precision	# Gold standard conserved complexes	# Detected conserved complexes	Recall (of conserved complexes)
COCIN	71	36	**50.7%**	42	32	**76.2%**

CMC	1202	145	12.1%	42	23	54.8%

HACO	1040	69	6.6%	42	17	40.5%

MCL	387	37	9.6%	42	5	11.9%

Predicted complexes: resulting network clusters

Matched predictions: resulting network clusters that match with benchmarks

Precision = #matched prediction/#predicted complexes

Recall = # detected conserved complexes/# gold standard conserved complexes

**Table 6:** Comparisons of different methods on human data

Method	# Predicted complexes	# Matched predictions	Precision	# Gold standard conserved complexes	# Detected conserved complexes	Recall (of conserved complexes)
COCIN	71	36	**50.7%**	118	78	**66.1%**

CMC	1389	156	11.2%	118	66	55.9%

HACO	1290	80	6.2%	118	36	30.5%

MCL	631	45	7.1%	118	24	20.3%

Predicted complexes: resulting network clusters

Matched predictions: resulting network clusters that match with benchmarks

Precision = #matched prediction/#predicted complexes

Recall = # detected conserved complexes/# gold standard conserved complexes

One predicted complex of COCIN can match with many benchmark complexes, this explains for #detected conserved complexes > #matched predictions (as illustrated in Figures 5-8)

**Figure 4:**
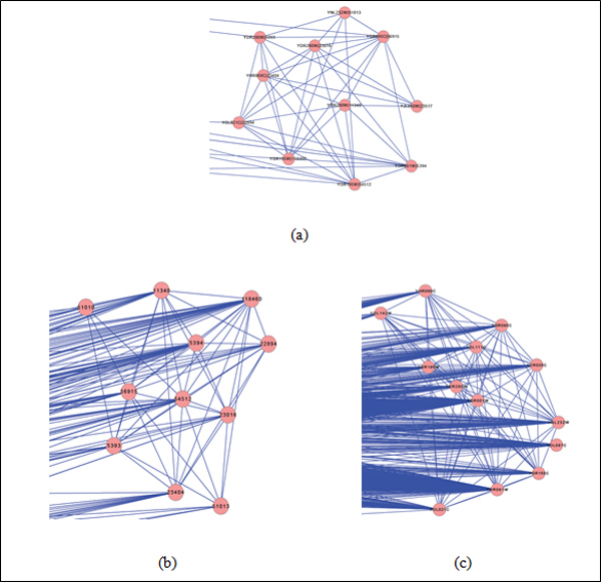
**An illustration on a predicted complexes from IN**. (a) A predicted complex in the IN. (b) The corresponding complex in the human PPI network. (c) The corresponding complex in the yeast PPI network.

Figure 4 compares a predicted complex *C_i _*through COCIN with two predictions *C_y _*and *C_h_*from the original PPI networks; *C_y _*and *C_h_*form a pair of orthologous complexes, but by direct clustering of the original PPI networks and matching them and not using COCIN. We noticed that *C_y _*and *C_h_*contained several noisy proteins and interactions among them which were false positives. These false positives reduced the Jaccard accuracy of these complexes when matched to known benchmark complexes. We also note that when we computed the complex-derivability index called *Component-Edge score *(this index measures how much of chance a complex can be detected given the topology of a PPI network) proposed in [24], *C_i _*had a higher CE-score compared to *C_y _*and *C_h _*in the networks.

**Figure 5:**
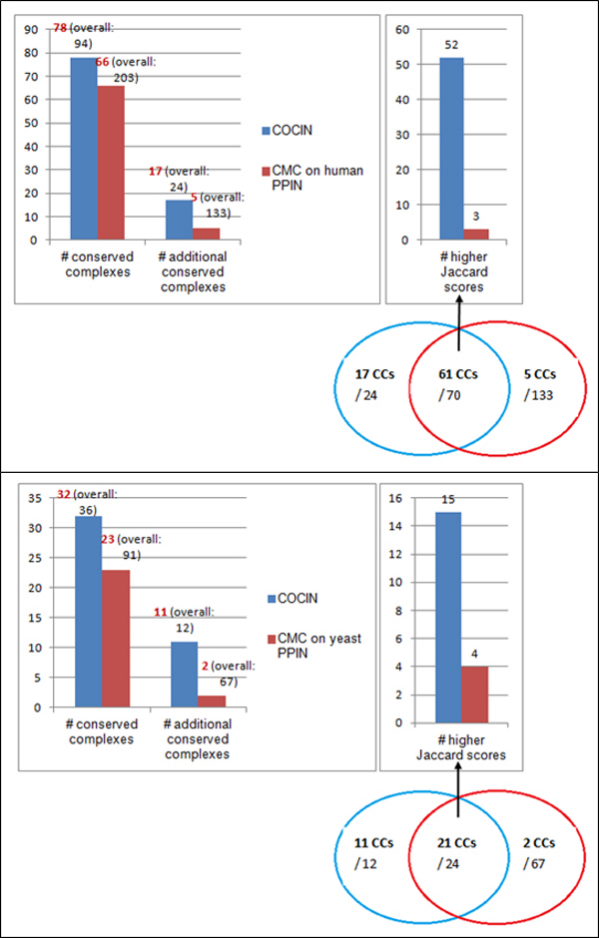
**COCIN compared to CMC**. COCIN over the interolog network identifies significantly more conserved complexes compared to direct clustering of the original PPI networks using CMC [19].

Figure 5 highlights the improvement of COCIN over CMC, that is, the additional protein complexes of human and yeast detected by COCIN. As many noisy interactions are removed in the IN, among the conserved complexes that are detected by both CMC and COCIN, COCIN on an average obtained higher Jaccard scores. Some important additional conserved complexes found using COCIN were: RNA Polymerase II, EIF3 complex, MSH2-MLH1-PMS2-PCNA DNA-repair initiation complex, MCM complex, MMR complex, Ubiquitin E3 ligase, transcription factor TFIID, DNA replication factor C, 20S proteasomes (descriptions of these complexes are listed in Tables 7 and 8).

**Table 7:** Additional conserved complexes found in yeast

ID	Complex name	Size	Jaccard score	Functional category	Functional description
96	eIF3 complex	7	0.63	Translation	Eukaryotic translation initiation factor

247	Transcription factor TFIID complex	15	0.73	Transcription	mRNA synthesis

27	DNA-directed RNA polymerase II complex	12	0.69	Transcription	mRNA synthesis

45	DNA replication factor C complex (Rad24p)	5	0.67	DNA processing	DNA synthesis and replication

152	DNA replication factor C complex (Rcf1p)	5	0.67	DNA processing	DNA synthesis and replication

294	Mcm2-7 complex	6	0.6	DNA processing	Chromosome maintainance, DNA synthesis and replication

268	SF3b complex	6	0.57	RNA processing	mRNA splicing

65	U6 snRNP complex	8	0.5	RNA processing	This complex combines with other snRNPs, unmodified pre-mRNA, and various other proteins to assemble a spliceosome, a large RNA-protein molecular complex upon which splicing of pre-mRNA occurs.

375	AP-3 adaptor complex	4	0.67	Cellular transport, vesicular transport	This complex is responsible for protein trafficking to lysosomes and other related organelles.

25	20S proteasome	14	0.5	Cell cycle, protein fate	Proteasomal degradation (ubiquitin/proteasomal pathway), protein processing (proteolytic)

137	Chaperonin-containing T-complex	8	0.67	Protein fate	A multisubunit ring-shaped complex that mediates protein folding in the cytosol without a cofactor.

**Table 8:** Additional conserved complexes found in human

ID	Complex name	Size	Jaccard score	Functional category	Function description
4392	EIF3 complex (EIF3A, EIF3B, EIF3G, EIF3I, EIF3C)	5	0.57	Translation	Translation initiation

4403	EIF3 complex (EIF3A, EIF3B, EIF3G, EIF3I, EIF3J)	5	0.57	Translation	Translation initiation

104	RNA polymerase II core complex	12	0.69	Transcription	mRNA synthesis

2685	RNA polymerase II	17	0.59	Transcription	mRNA synthesis

2686	BRCA1-core RNA polymerase II complex	13	0.64	Transcription	mRNA synthesis

471	PCAF complex	10	0.6	Transcription, DNA processing	DNA conformation modification (e.g. chromatin), modification by acetylation, deacetylation, organization of chromosome structure.

2200	RFC2-5 subcomplex	4	0.5	DNA processing	DNA synthesis and replication

387	MCM complex	6	0.6	DNA processing	Chromosome maintainance, DNA synthesis and replication

369	MMR complex 2	4	0.67	DNA processing	DNA damage repair

290	MSH2-MLH1-PMS2-PCNA DNA-repair initiation complex	4	0.67	DNA processing	DNA damage repair initiation

1169	SNARE complex	4	0.6	Cellular transport, vesicular transport	Vesicle fusion, synaptic vesicle exocytosis

562	LSm2-8 complex	7	0.67	RNA processing	mRNA splicing

561	LSm1-7 complex	7	0.67	RNA processing	Control of mRNA stability during splicing

3036	Ubiquitin E3 ligase (SKP1A, SKP2, CUL1, CKS1B, RBX1)	5	0.5	Cell cycle, protein fate	Mitotic cell cycle and cell cycle control, modification by ubiquitination, deubiquitination

2188	Ubiquitin E3 ligase (CDC34, NEDD8, BTRC, CUL1, SKP1A, RBX1)	5	0.5	Cell cycle, protein fate	Mitotic cell cycle and cell cycle control, modification by ubiquitination, deubiquitination

2189	Ubiquitin E3 ligase (SMAD3, BTRC, CUL1, SKP1A, RBX1)	5	0.5	Cell cycle, protein fate	Mitotic cell cycle and cell cycle control, modification by ubiquitination, deubiquitination

### The result of complex detection in the conserved subnetworks

To further understand the advantage of COCIN on leveraging conservation for better detection of complexes, we performed another experiment *alternative *to the interolog network as follows. We predicted complexes from the *subset of protein interactions of the first species that are conserved in the second *(we call this the *conserved subnetwork *in the first species). However, this can only find complexes of one species at a time, so we map these predicted complexes onto the PPI network of the other species to identify the corresponding conserved complexes. We employed CMC to do clustering on the conserved subnetworks.

Complex prediction from conserved subnetworks showed similar result as COCIN -16 additional conserved complexes in human and 9 additional conserved complexes in yeast are found. This supported the purpose of IN - to leverage conserved interactions for improving complex prediction.

**Figure 6:**
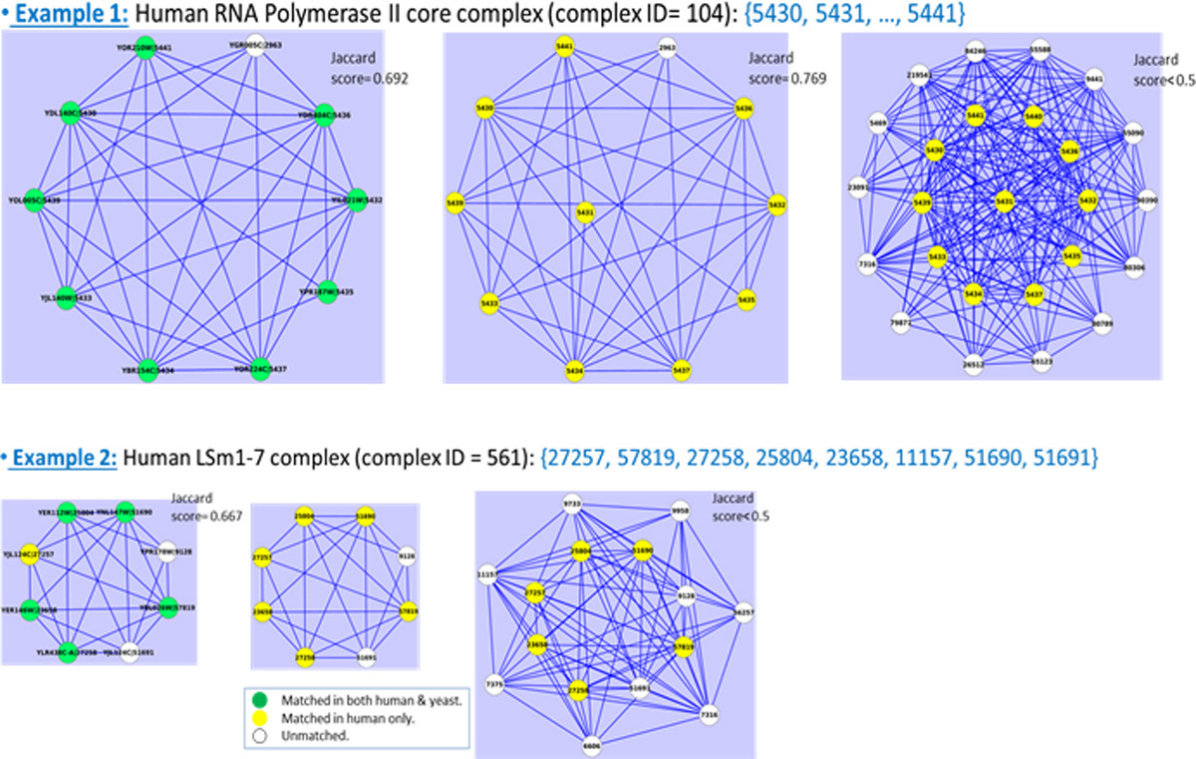
**Some examples of additional conserved complexes found in IN**. The clusters detected from the original PPI networks include several noisy proteins and noisy interactions (false positives), thereby reducing their Jaccard accuracies.

Figure 6 shows two other examples that explain why additional conserved complexes are found by COCIN but missed by CMC. We see from this picture that the predicted human complex from IN (the leftmost figure) and the corresponding predicted complex from the conserved subnetwork (the center figure) were contained in a *larger *CMC-predicted complex (the rightmost figure) from the original PPI networks. This larger complex included several noisy proteins that reduce the accuracy of the complex, thereby causing the complex to be missed.

### Comparisons with other complex detection methods in PPI networks

**Figure 7:**
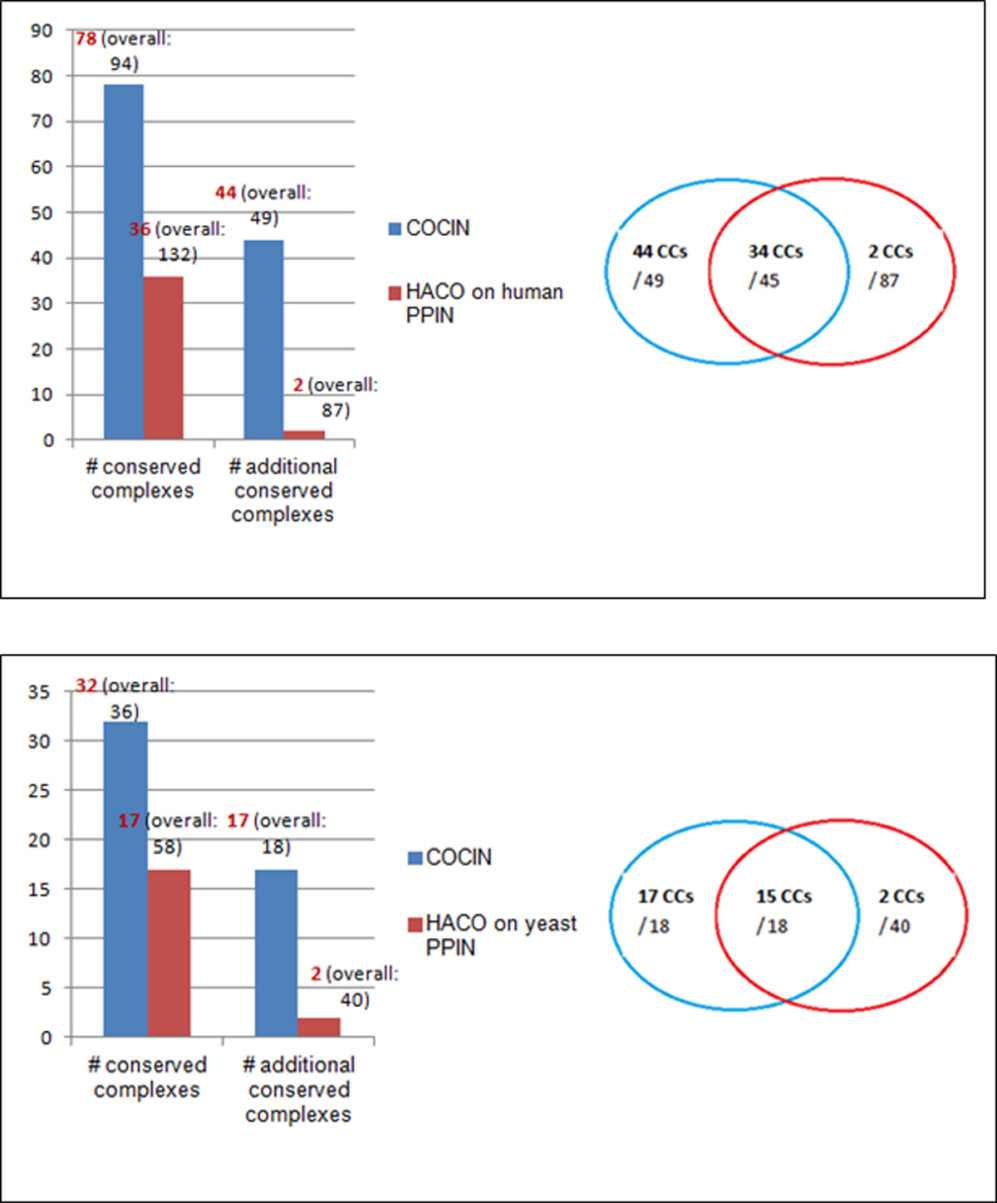
**COCIN compared to HACO**. COCIN over the interolog network identifies significantly more conserved complexes compared to direct clustering of the original PPI networks using HACO [20].

**Figure 8:**
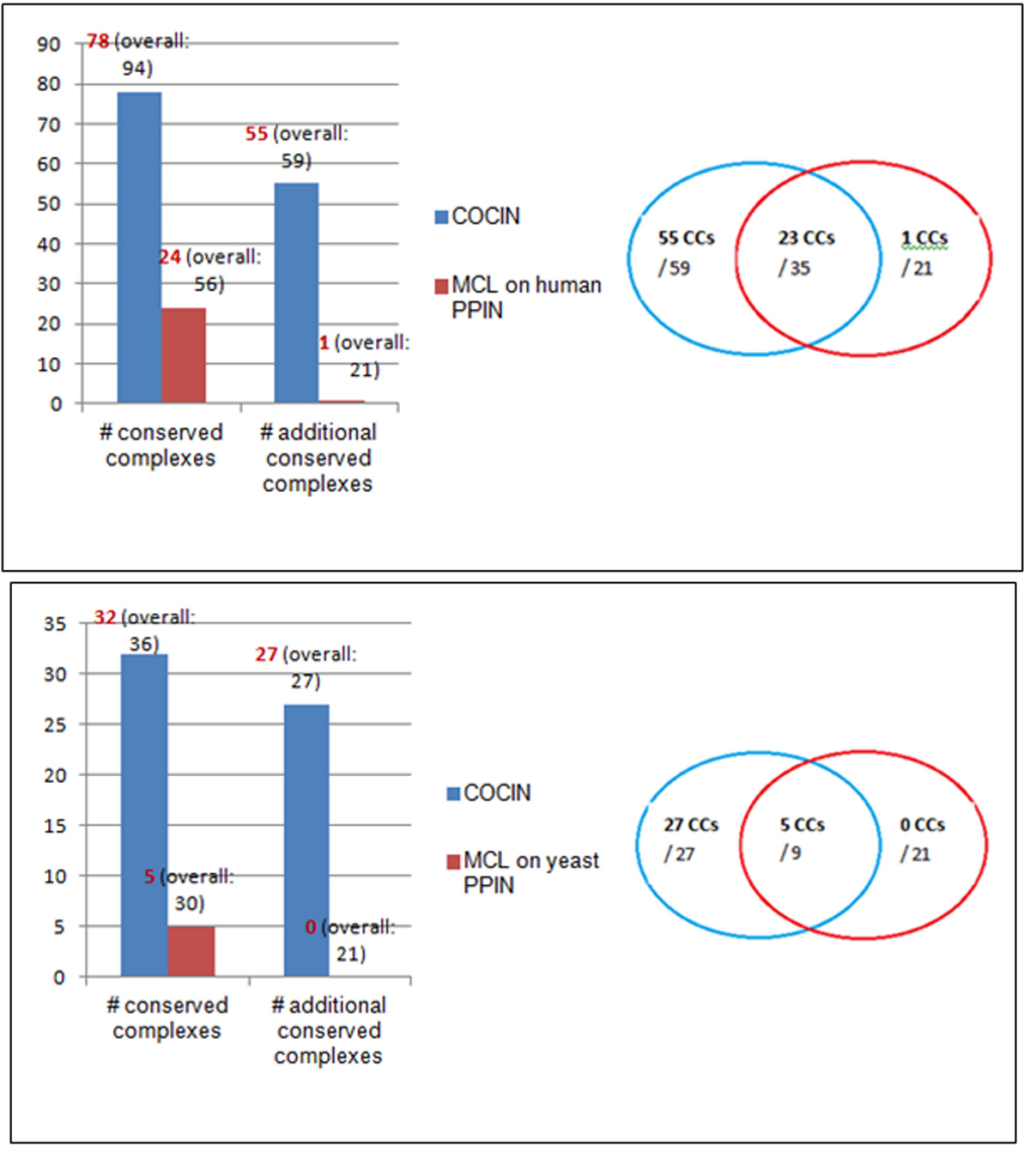
**COCIN compared to MCL**. COCIN over the interolog network identifies significantly more conserved complexes compared to direct clustering of the original PPI networks using MCL [21].

Similar results were obtained using the other two methods HACO and MCL as well, thereby supporting the effectiveness of COCIN in identifying conserved protein complexes. Tables 5 and 6 present these comparisons in more details, while Figures 7 and 8 highlight further substantiate these results.

### Integrating domain information significantly enhances interolog construction

**Figure 9:**
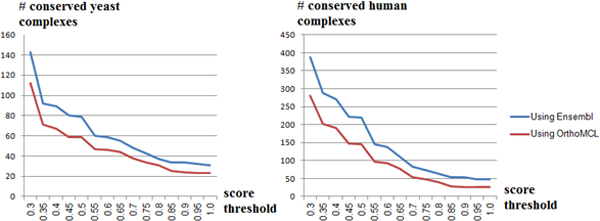
**Assessment of Ensembl and OrthoMCL based homology for IN construction and conserved-complex detection**. Ensembl [17] contains protein orthologs based on sequence similarity as well as domain information, while OrthoMCL [18] is predominantly based on sequence similarity. As we can see from the table, using domain information (through Ensembl) generates significantly more many-to-many ortholog mappings thereby enhancing our interolog construction.

**Figure 10:**
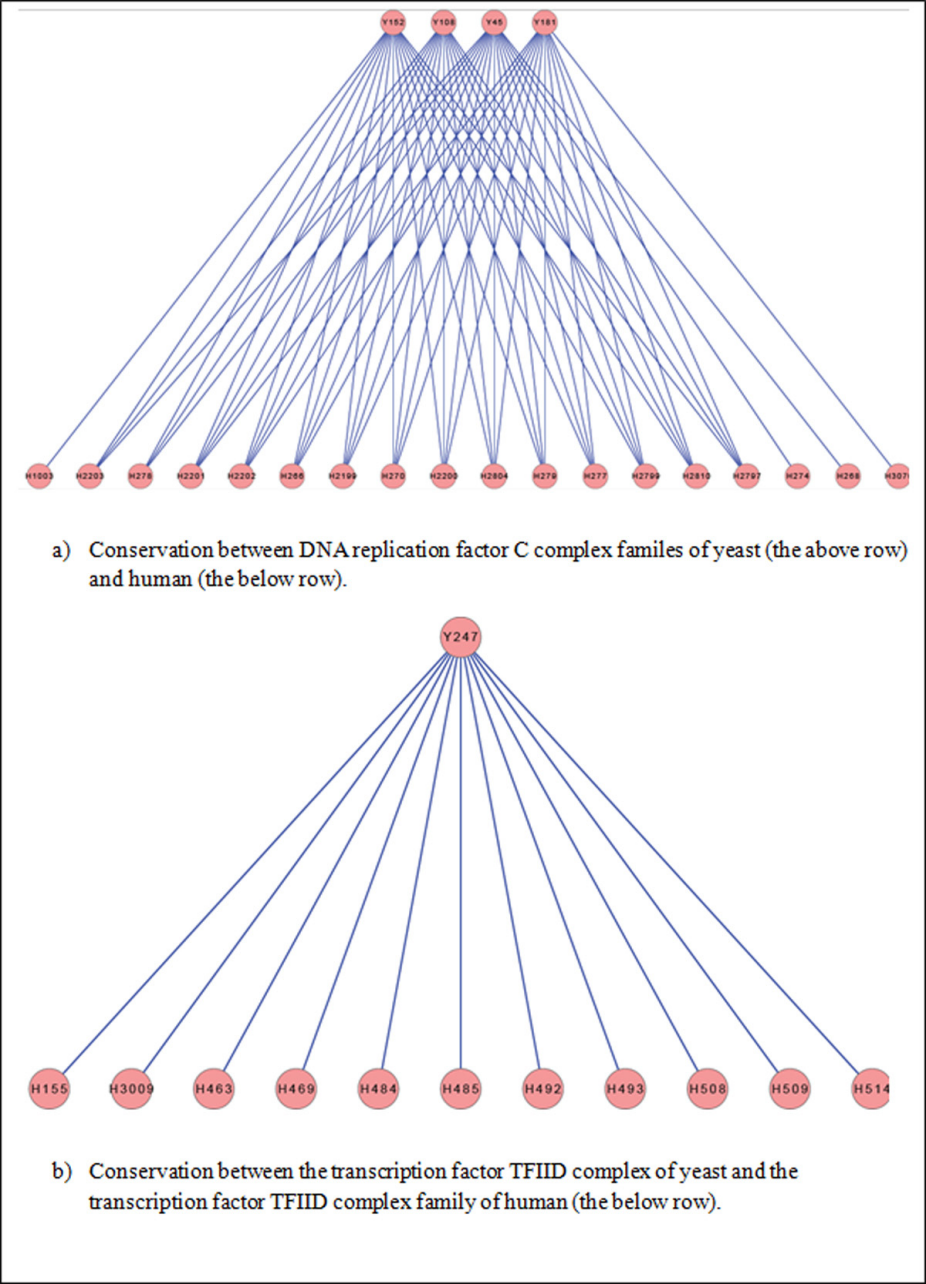
**Some examples of the one-to-many and many-to-many relationships of complex conservation between human and yeast**. Ensembl [17] contains protein orthologs based on sequence similarity as well as domain information, while OrthoMCL [18] is predominantly based on sequence similarity. As we can see from the table, using domain information (through Ensembl) generates significantly more many-to-many ortholog mappings thereby enhancing our interolog construction.

Finally, Table 9 summarizes the quality of our testing dataset for conserved protein complexes between yeast and human. We compared the number of benchmark conserved complexes found in both human and yeast using mappings from Ensembl and OrthoMCL under multiple conservation score thresholds (Figure 9). *Note *that Ensembl contains homology information based on both sequence similarity as well as domain conservation, while OrthoMCL is predominantly based on sequence similarity. We noticed that using Ensembl homology information can yield more conserved complexes at all conservation score thresholds. Further, Figure 10 shows that there exist 1-to-many and many-to-many relationships of conservation between human and yeast complexes.

**Table 9:** Details of gold standard testing dataset for conserved protein complexes between human and yeast

Score usage	*MSJ*≥threshold
Threshold	50%

# conserved *yeast *complexes	**42**/149 with size>3 (*28.1%*)
	
	Total: 79/408 (19.3%)

# conserved *Human *complexes	**118**/722 with size>3 (*16.3%*)
	
	Total: 219/1843 (11.9%)

**Figure 11:**
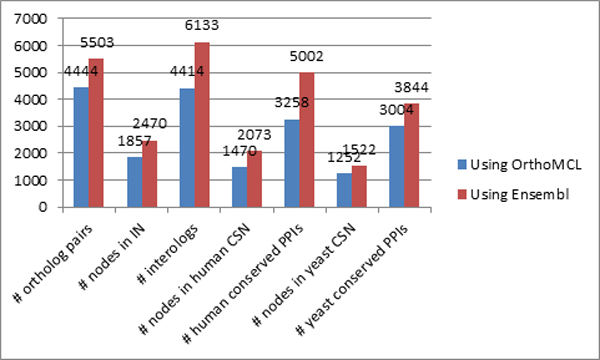
**Comparison between using Ensembl and OrthoMCL in constructing the interolog network**. Ensembl [17] contains protein orthologs based on sequence similarity as well as domain information, while OrthoMCL [18] is predominantly based on sequence similarity. As we can see from the table, using domain information (through Ensembl) generates significantly more many-to-many ortholog mappings thereby enhancing our interolog construction.

Sharan et al. used whole-sequence similarity to construct the interolog network. Here, we used OrthoMCL as a substitute for the whole-sequence similarity due to technical difficulties of running BLAST for a large number of proteins. We compared the performance of using OrthoMCL against using Ensembl, which uses domain conservation along with sequence similarity to determine orthology. Table 10 and Figure 11 show that we obtain an overall improvement in terms of the number of mapped protein pairs, interologs, as well as conserved protein complexes in both human and yeast by incorporating domain information (through Ensembl). This substantiates the improved performance of COCIN over traditional sequence-similarity based methods.

**Table 10:** Homology data: Ensembl and OrthoMCL

		Ensembl database	OrthoMCL database
# Ortholog groups:	# 1-to-1 groups	1096	1153
	
	# 1-Yeast-to-many groups	756	434
	
	# 1-Human-to-many groups	116	116
	
	# many-to-many groups	197	167
	
	Total:	**2165 **(5503 pairs)	**1870**

# Human paralog groups:		2573	2435

# Yeast paralog groups:		426	393

Total # homolog groups:		5164	4698

Ensembl [17] contains protein orthologs based on sequence similarity as well as domain information, while OrthoMCL [18] is predominantly based on sequence similarity. As we can see from the table, using domain information (through Ensembl) generates significantly more many-to-many ortholog mappings thereby enhancing our interolog construction.

## Discussion

Figure 1 paints a very complicated picture for the conservation pattern of protein complexes from yeast to human. We believe that this picture reflects the actual situation, and it overrides the belief that a yeast complex is essentially a (proper) subset of a human complex with only a few new proteins added to the human complex. In other words, the conservation pattern from yeast to human is highly intricate involving dispersion and re-distribution of proteins and their functions across complexes along with addition of several new proteins in human. At a very high level, though core cellular mechanisms such as cell cycle and DDR are indeed conserved from yeast to human, within these mechanisms, considerable re-arrangements have occurred. This finding can have implications on studies attempting to extrapolate relationships such as synthetic lethality (SL) from yeast to human. In particular, we believe that many of the SL relationships may not be conserved from yeast to human, or even if conserved, may not be identifiable by simple BLAST-based sequence-similarity mappings.

## Conclusions

Identifying conserved complexes between species is a fundamental step towards identification of conserved mechanisms from model organisms to higher level organisms. Current methods based on clustering PPI networks do not work well in identifying conserved complexes, and they are severely limited by lack of true interactions and presence of large amounts of false interactions in existing PPI datasets. Here, we presented a method COCIN based on building interolog networks from the PPI networks of species to identify conserved complexes. Our experiments on yeast and human datasets revealed that our method can identify considerably more conserved complexes that plain clustering of the original PPI networks. Further, we demonstrated that integrating domain information generates many-to-many ortholog relationships which significantly enhances interolog quality and throws further light on conservation of mechanisms between yeast and human.

## Availability

Our COCIN software and the datasets used in this work are freely available at: http://www.comp.nus.edu.sg/~leonghw/COCIN/ or alternately at: https://sites.google.com/site/mclcaw/. The preliminary version of this work appeared as a poster paper (abstract) at RECOMB 2013 [30].

## Competing interests

The authors declare that they have no competing interests.

## Authors' contributions

All the authors have equal contributions to the ideas of the work. PVN performed the experiments and analysis, software development. SS and PVN took part in writing the manuscript. HWL supervised the project and reviewed the manuscript. All the authors have read and approved the manuscript.

## Declarations

Publication of this article was funded by an NUS Singapore ARF grant R252-000-461-112, and SS was supported by an Australian NHMRC grant 1028742 to Dr Peter T. Simpson and Professor Mark A. Ragan.

This article has been published as part of *BMC Bioinformatics *Volume 14 Supplement 16, 2013: Twelfth International Conference on Bioinformatics (InCoB2013): Bioinformatics. The full contents of the supplement are available online at http://www.biomedcentral.com/bmcbioinformatics/supplements/14/S16.
